# *Mycenabrunnescens* (Basidiomycota, Mycenaceae), a new species of *Mycena* sect. *Pterigenae* from China

**DOI:** 10.3897/BDJ.12.e125570

**Published:** 2024-07-25

**Authors:** Hong Zhang, Ying Xin Xiao, Zhu Ming Tan, Ai Rong Shen, Bao Ming Shen, Yun Tan, Sai Nan Li, Li Guo Feng, Zhu Xiang Liu, Li Na Liu

**Affiliations:** 1 College of Biology and Environmental Sciences, Jishou University, Jishou 416000, China College of Biology and Environmental Sciences, Jishou University Jishou 416000 China; 2 Hunan Academy of Forestry, Changsha 410004, China Hunan Academy of Forestry Changsha 410004 China; 3 Hunan Edible Fungi Research Institute, Changsha 410004, China Hunan Edible Fungi Research Institute Changsha 410004 China

**Keywords:** new taxon, molecular phylogeny, taxonomy

## Abstract

**Background:**

*Mycena* (Pers.) Roussel (1806) is a large genus of Mycenaceae known for having small to medium-sized basidiomata. It is typified by the species *Mycenagalericulata* (Scop.) Gray. For years, many mycologists have made important contributions to understanding *Mycena* and several monographs have been published. Three specimens were collected from China that belonged to the genus *Mycena*. On the basis of morphological analysis and phylogenetic analyses employing DNA sequences, a new species is described.

**New information:**

*Mycenabrunnescens* sp. nov. is described as a new species from subtropical areas of China. It is characterised by its brown pileus, whitish lamellae that turns brown when bruised, orange to brown lamellae edges, the absence of pleurocystidia and cheilocystidia with simple or branched excrescences at the apex containing yellowish-brown contents. We performed phylogenetic analyses on a concatenated dataset comprising the internal transcribed spacer and large subunit regions of nuclear ribosomal RNA using Bayesian Inference and Maximum Likelihood methods. The result showed that the new taxon clustered in an independent group and is closely related to *M.albiceps* and *M.flosoides*.

## Introduction

*Mycena* (Pers.) Roussel (Roussel 1806) (Mycenaceae, Agaricales) is a large genus composed of at least 500 species worldwide (Kirk et al. 2008). *Mycena* species are known for their small to medium-sized basidiomata. Some *Mycena* species were reported to be bioluminescent, emitting light through their basidiomata, hyphae or both (Chew et al. 2014, Chew et al. 2015, Cooper et al. 2018, Cortés-Pérez et al. 2019, Desjardin et al. 2007, Desjardin et al. 2010). Additionally, *Mycena* can play a vital role in ecology by participating in the decomposition process of organic matter; moreover, some serve as the germination fungi for *Gastrodiaelata* seeds (Frankland 1998, Liu et al. 2022b).

China has abundant *Mycena* resources and many new *Mycena* species have been recorded to date (Ge et al. 2021, Liu et al. 2021, Liu et al. 2022, Na and Bau 2018, Na and Bau 2019a, Na and Bau 2019b, Na et al. 2022, Qiang and Bai 2023, Wei et al. 2024). During our field investigations of mycenoid fungi in China, we discovered a new species. Detailed morphological features and phylogenetic analysis, based on the ITS and LSU sequences, indicate that the new taxon is distinct from morphologically similar and phylogenetically related species.

Based on the morphological classification proposed by Maas Geesteranus (Maas Geesteranus 1992), this new taxon belongs to sect. Pterigenae. Section Pterigenae was initially classified into the subsect. Pterigenae of sect. Luculentae. Maas Geesteranus later discovered the only species of the subsect. Pterigenae, *M.pterigena*, lacks pleurocystidia, which is different from other species in sect. Luculentae. Therefore, Mass Geesteranus elevated it to section rank (Robich 2016). Mycenasect.Pterigenae (Maas G.) Maas G. is characterised by an orange-red to pink pileus and lamellae edges, cheilocystidia typically covered with cylindrical excrescences containing coloured contents, absence of pleurocystidia, hyphae of the pileipellis and stipitipellis densely ornamented with warts (Maas Geesteranus 1986). It currently comprises two taxa, *Mycenapterigena* (Fr.) P. Kumm. and *Mycenacapillofasciculata* Robich.

## Materials and methods

### Sampling, morphological observations and descriptions

Specimens in this study were collected from Guangxi Zhuang Autonomous Region and Sichuan Province, dried with silica gel and deposited in the Herbarium of Jishou University (JIU). Macroscopic characters were described by field observations and digital images, with colour terms following Kornerup and Wanscher (1978). Microscopic characters were observed on dried specimens under light microscopy (Olympus BX51) and specimens were mounted in pure water and 5% potassium hydroxide (KOH) or 1% Congo red. Melzer’s reagent was used to test the amyloidity of basidiospores and dextrinoid reaction of tissues. Thirty spores were measured per basidioma with Q being the ratio of basidiospore length to its width in side view. Other microscopic features required at least 20 measurements from each specimen.

### DNA extraction, PCR amplification and sequencing

Total genomic DNA was extracted using the NuClean Plant Genomic DNA kit (CWBIO, Norcross, GA) according to the manufacturer’s instructions. The internal transcribed spacer (ITS) and 28S large subunit regions of ribosomal DNA were amplified with the primer pairs ITS5/ITS4 and LR0R/LR7 (White et al. 1990). PCR conditions for ITS and LSU followed (Zhang et al. 2019) and the amplified PCR products were purified and sequenced by Sangon Biotech (Shanghai, China) for purification and sequencing.

### Data analyses

For molecular phylogenetic analyses of the combined dataset (ITS+LSU), the sequences were aligned using MAFFT v.7.310 (Katoh and Standley 2013) and manually edited using BioEdit v.7.0.5 (Hall 1999). In the alignment, gaps were treated as missing data. MrModelTest v.2.3 was used to determine the best fit model, based on the Akaike Information Criterion (Nylander 2004). Maximum Likelihood (ML) analysis was performed using RAxML-NG v.0.9.0 with 1000 bootstrap replicates (Kozlov et al. 2019) and Bayesian Inference (BI) analysis was performed using MrBayes 3.2.6 (Ronquist and Huelsenbeck 2003). The analysis ran for 1,0000,000 MCMC generations with four chains, sampling every 1,000 generations, the initial 25% of sampled data being discarded as burn-in. Phylogenetic trees were visualised with FigTree v.1.4.3. The outgroup selected was from Liu et al. (2022a).

## Taxon treatments

### Mycena brunnescens

L.N. Liu sp. nov.

1782FDD3-2F3E-5001-B6F7-C65E129B6423

851945

#### Materials

- **Type status:** Holotype. **Occurrence:** occurrenceID: 2723CE40-21C3-5048-93D7-89379C9BF8F0; **Taxon:** kingdom: Fungi; phylum: Basidiomycota; class: Agaricomycetes; order: Agaricales; family: Mycenaceae; genus: Mycena; taxonRank: species; **Location:** country: China; stateProvince: Guangxi; county: Leye; verbatimLocality: Yachang Orchid National Nature Reserve; verbatimLatitude: 24^◦^29′04.62″ N; verbatimLongitude: 106^◦^22′35.40″ E; **Identification:** identifiedBy: Ying Xin Xiao; **Event:** eventDate: 30 June 2021; **Record Level:** institutionID: JIU; collectionID: JIU125
- **Type status:** Paratype. **Occurrence:** occurrenceID: 10688CAA-8D2B-56EE-86AD-CF320C05CC3E; **Taxon:** kingdom: Fungi; phylum: Basidiomycota; class: Agaricomycetes; order: Agaricales; family: Mycenaceae; genus: Mycena; taxonRank: species; **Location:** country: China; stateProvince: Guangxi Zhuang Autonomous Region and Sichuan Province; county: Leye; verbatimLocality: Yachang Orchid National Nature Reserve; verbatimLatitude: 27°23′40′′ N; verbatimLongitude: 106°11′35′′ E; **Identification:** identifiedBy: Ying Xin Xiao; **Event:** eventDate: 30 June 2021; **Record Level:** institutionID: JIU; collectionID: JIU126
- **Type status:** Paratype. **Occurrence:** occurrenceID: 611F3C05-18F0-5409-A51F-6496001496C9; **Taxon:** kingdom: Fungi; phylum: Basidiomycota; class: Agaricomycetes; order: Agaricales; family: Mycenaceae; genus: Mycena; taxonRank: species; **Location:** country: China; stateProvince: Sichuan province; **Identification:** identifiedBy: Ying Xin Xiao; **Event:** eventDate: 30 September 2023; **Record Level:** institutionID: JIU; collectionID: JIU127

#### Description

**Figure 1.**
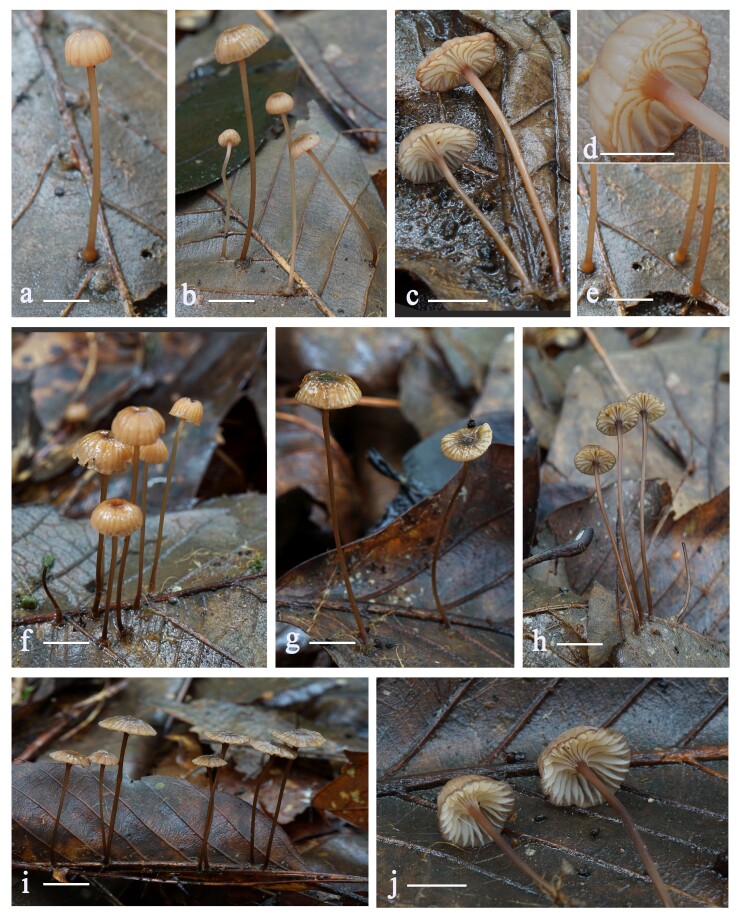
Basidiomata of *Mycenabrunnescens*. Scale bar = 5 mm. Photos by Li Na Liu.

Pileus 3–8 mm diam., hemispherical, plane-convex to nearly applanate, umbonate to deppresed to almost subumbilicate, first translucent-striate, then sulcate, glabrous, light orange (6A5) to orange (6B7) when young, becoming yellowish-brown (6F6) or dark brownish-grey (6F8) in the disc and in the grooves with age, margin concolorous or paler, pale yellowish-brown (5D8) to brown (6D7) or dark brown (6F4–6F8). Context thin, fragile, whitish. Lamellae decurrent, moderately distant (L = 15–20, I = 1–2), changing from whitish (1A1) to dark brown (6F7–6F8) when bruised, lamellae edges light orange to orange (6A5-6B7), light brown (6D4–6D8) to brown (6E8). Stipe 17–34 × 1–2 mm, cylindrical, hollow, surface smooth, yellowish-red (8B6) to reddish-brown (8D6) towards apex when young, becoming yellowish-brown (6F6–6F8) in age, the upper portion brownish-orange (6C6), light brown (6D4–6D8) or brown (6E4–6E8), equal and with a slightly bulbous base, covered with whitish fibrils (Fig. 1). Odour and taste not distinctive.

**Figure 2.**
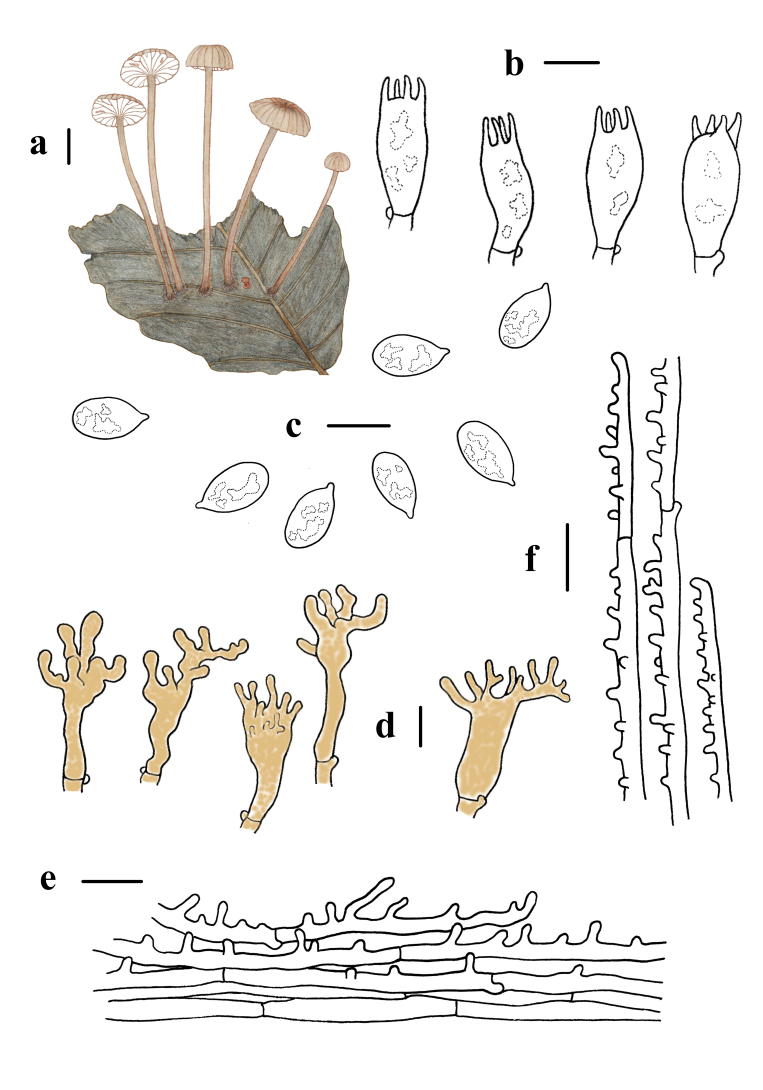
Morphological features of *Mycenabrunnescens*. **a** basidiomata; **b** basidia; **c** basidiospores; **d** cheilocystidia; **e** pileipellis; **f** stipitipellis. Scale bars: a = 5 mm, b, c, d, e, f = 10 μm.

Basidiospores 5.9–7.3 (7.5) × (3.1) 3.2–3.8 μm, Q = 1.6–2.2, ellipsoid to oblong, few subcylindrical, smooth, hyaline, amyloid, thin-walled. Basidia 14–23 × 5–10 μm, short clavate or clavate, 4–spored, thin walled. Cheilocystidia 20–42 × 6–12 μm, clavate or cylindrical, with branches excrescences at the apex, 3–10 × 1–3 μm, with yellowish- brown (5D8) contents. Pleurocystidia absent. Pileipellis a cutis, hyphae of the pileipellis 1.6–4 μm wide, hyaline, densely covered with cylindrical excrescences, 1–4 × 1–2 μm. Hyphae of the stipitipellis 1–7 μm wide, with cylindrical excrescences 1–4 × 1–2 μm, hyaline, thin-walled. Clamps present in all tissues (Fig. 2).

#### Diagnosis

*Mycenabrunnescens* has a brown pileus, lamellae that change from whitish to brown when bruised, orange to brown lamellae edges, basidiospores ellipsoid to oblong, cheilocystidia clavate with yellowish-brown contents, pileipellis and stipitipellis covered with cylindrical excrescences. Differs from *M.strobilinoidea* by branched cheilocystidia and absent pleurocystidia.

#### Etymology

Referring to the colour of basidiomata.

#### Distribution

Only known from Guangxi Zhuang Autonomous Region and Sichuan Province.

#### Ecology

Scattered or gregarious on decayed leaves.

## Analysis

### Phylogenetic analyses

**Figure 3.**
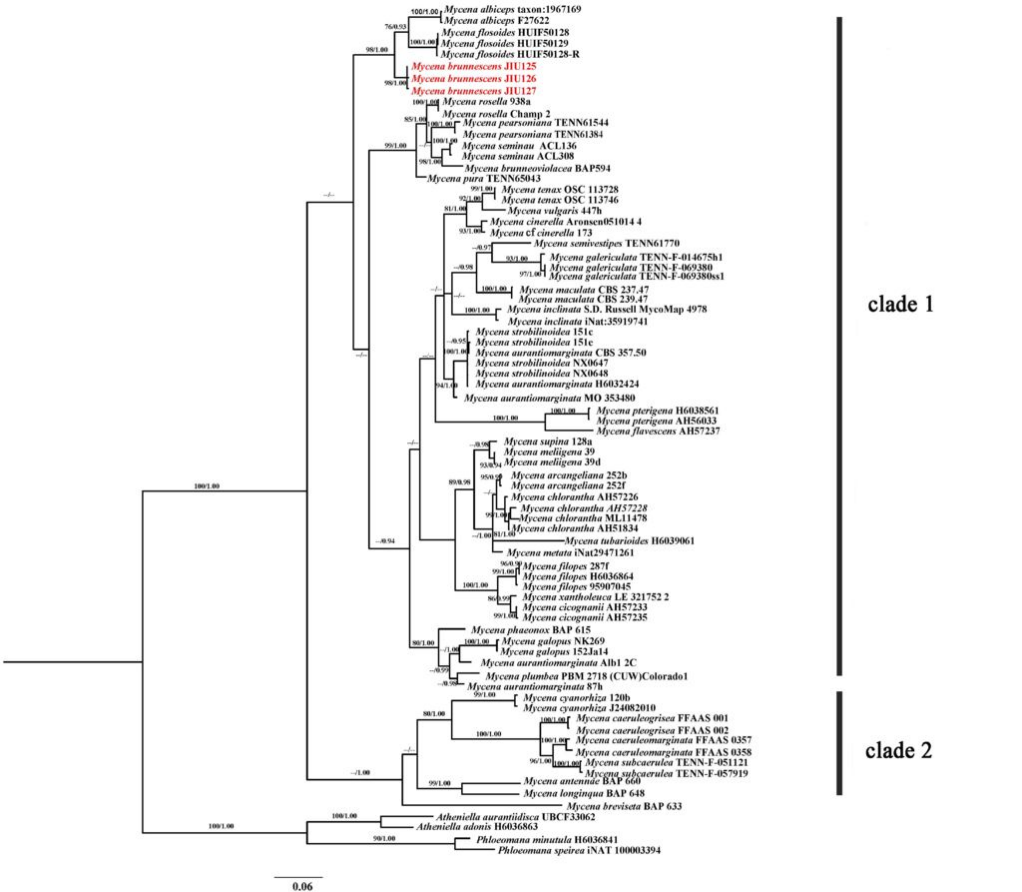
Bayesian tree inferred from ITS and LSU sequences showing phylogenetic relationships of *Mycenabrunnescens*. Bayesian Inference (≥ 0.90) and Maximum Likelihood support values (≥ 75) are indicated above the branches.

A total of 94 sequences (ITS and LSU) were used for phylogenetic reconstruction, including five sequences generated in this study and 89 sequences retrieved from GenBank. Sequences selection was mainly based on similar morphological characteristics, a BLAST result and previous research (Table 1). Based on the optimal evolutionary model selected for ITS and LSU sequences GTR+I+G, ML and BI trees shared similar topology. Thus only the BI tree is presented (Fig. 3).

The phylogenetic tree contained two major clades. The new species was placed in Clade 1. *Mycenapterigena*, which belongs to the same section, was also placed in Clade 1, but showed a distant relationship with the new species. The new taxon from China formed a monophyletic lineage (ML/PP = 98/1.00) and grouped with *M.flosoides* L. N. Liu and *M.albiceps* (Peck) Gilliam forming a small branch with high statistical support value (ML/PP = 98/1.00). Therefore, the new taxon can be clearly separated from *M.flosoides* and *M.albiceps* (Fig. 3).

## Discussion

*Mycenabrunnescens* is characterised by its brown pileus, decurrent lamellae, whitish lamellae that change to brown when bruised, lamellae edges orange to brown, a smooth stipe with a slightly enlarged base, cheilocystidia clavate, covered with simple to branched, cylindrical excrescences, and containing yellowish-brown contents, pileipellis and stipitipellis covered with simple and scattered excrescences. It belongs to sect. Pterigena, based on its brightly coloured basidiomata and lamellae edges, cheilocystidia with long excrescences and absence of pleurocystidia, hyphae of pileipellis and stipitipellis with excrescences (Maas Geesteranus 1986, Robich 2016). Two species, *M.capillofasciculata* and *M.pterigena* are currently in this section. It is worth mentioning that *M.pterigena* has been reported in China (Wang 2013). However, *M.pterigena* can be easily distinguished from *M.brunnescens* by its pink pileus and stipe, pink lamellae edges, typically occurring on decaying fern stalks and longer basidiospores, longer and unbranched cylindrical excrescences on the apex of the cheilocystidia and pileipellis with terminal cells similar to cheilocystidia (Maas Geesteranus 1986, Robich 2003, Robich 2016, Uzun and Demirel 2017). *Mycenacapillofasciculata* was originally described from Italy by Robich. It differs by its light pink and pale brownish-pink pileus, deep rose lamellae edges, smooth cheilocystidia or few excrescences, stipe with long fibrils, united in bundles and broader basidiospores (Robich 2016). Mycenasect.Pterigena was initially assigned to subsect. Pterigenae of sect. Luculentae by Maas Geesteranus, with three species belonging to sect. Luculentae: *M.aurantiomarginata* (Fr.) Quél., *M.rosella* (Fr.) P. Kumm. and *M.strobilinoidea* Peck. Amongst them, *M.strobilinoidea* resembles the new species, but it can differ in its orange-yellow lamellae, reddish-orange lamellar edges and the presence of pleurocystidia (Maas Geesteranus 1980, Na and Bau 2018, Perry 2002). *Mycenabrunnescens* cannot be mistaken for the other species of sect. Luculentae because its lamellae gradually change from whitish to brown when bruised. In our phylogenetic analysis, ML and BI trees, based on ITS and LSU sequences, show that the three specimens of the new species were palced in a small branch of Clade 1. *Mycenapterigena* of the same section clustered in a different branch within Clade 1 and was sister to *M.flavescens* Velen.. Additionally, it can be clearly distinguished from this new taxon. *Mycenaflosoides* and *M.albiceps* were located in the same small branch with the new taxon. *Mycenaflosoides* differs by its pink pileus and lamellae, shorter basidia, cheilocystidia without coloured contents, pileipellis with terminal cells up to 42 × 16 μm and the presence of brown rhizomorphs (Liu et al. 2022a). In comparision, *M.albiceps* has a white pileus, black stipes with brown mycelium and longer stipes (Gilliam 1976, Liu et al. 2022a). Furthermore, the three species in the same small branch share some common characteristics, including the same basidiospore shapes and decurrent lamellae.

## Supplementary Material

XML Treatment for Mycena brunnescens

## Figures and Tables

**Table 1.** Specimens used in phylogenetic analysis and GenBank accession numbers.

Species	Voucher	GenBank Accession no.		Country
		ITS	LSU	
*Atheniellaadonis*	H6036863	MW540691	-	Finland
*Atheniellaaurantiidisca*	UBCF33062	MF908459	-	Canada
*Mycenaalbiceps*	F27622	MZ303026	-	USA
*Mycenaalbiceps*	taxon:1967169	MK234177	-	USA
*Mycenaanntennae*	BAP 660	MH414550	MH385326	São Tomé
*Mycenaarcangeliana*	252f	JF908402	-	Spain
*Mycenaarcangeliana*	252b	JF908401	-	Spain
*Mycenaaurantiomarginata*	Alb1-2C	MN328293	-	Argentina
*Mycenaaurantiomarginata*	87h	JF908479	-	Italy
*Mycenaaurantiomarginata*	H6032424	MW540657	-	Finland
*Mycenaaurantiomarginata*	MO 353480	MN202587	-	USA
*Mycenaaurantiomarginata*	CBS:357.50	MH856657	MH868173	France
*Mycenabreviseta*	BAP 633	MH414551	MH385327	Príncipe
*Mycenabrunneoviolacea*	BAP 594	MH414546	-	São Tomé
***Mycenabrunnescens***	**JSU125 (holotype)**	**ON778578**	**OP360941**	**China**
***Mycenabrunnescens***	**JSU126**	**ON778579**	**OP360942**	**China**
***Mycenabrunnescens***	**JSU127**	**PP152232**	-	**China**
*Mycenacaeruleogrisea*	FFAAS 0001	MW051896	OL711662	China
*Mycenacaeruleogrisea*	FFAAS 0002	MW051897	OL711663	China
*Mycenacaeruleomarginata*	FFAAS 0357	OL711669	OL711664	China
*Mycenacaeruleomarginata*	FFAAS 0358	OL711670	OL711665	China
Mycenacf.cinerella	173	MF926553	-	-
*Mycenachlorantha*	AH51834	OR141886	-	Spain
*Mycenachlorantha*	ML11478	OR141887	-	Spain
*Mycenachlorantha*	AH57228	OR141885	-	Spain
*Mycenachlorantha*	AH57226	OR141884	-	Spain
*Mycenacicognanii*	AH57233	OR141876	-	Spain
*Mycenacicognanii*	AH57235	OR141878	-	Spain
*Mycenacinerella*	Aronsen051014	KT900146	-	Norway
*Mycenafilopes*	95907045	ON175868	-	America
*Mycenafilopes*	H6036864	MW540692	-	Finland
*Mycenafilopes*	287f	JF908410	-	Italy
*Mycenaflavescens*	AH57237	OR141883	-	Spain
*Mycenaflosoides*	HUIF50128	OP358282	OP360939	China
*Mycenaflosoides*	HUIF50129	OP358283	OP360940	China
*Mycenaflosoides*	HUIF50128-R	OP745013	-	China
*Mycenagalericulata*	TENN-F-069380ss1	MN088383	-	USA
*Mycenagalericulata*	TENN-F-069380	MN088382	-	USA
*Mycenagalericulata*	TENN-F-014675h1	MN088380	-	USA
*Mycenagalopus*	NK269	FR846482	-	Czech Republic
*Mycenagalopus*	152Ja14	KU516420	-	Poland
*Mycenagreen-blueorhiza*	J24082010	MW540696	-	Finland
*Mycenagreen-blueorhiza*	120b	JF908385	-	Italy
*Mycenainclinata*	S.D. Russell MycoMap 4978	MK532829	-	USA
*Mycenainclinata*	iNat:35919741	MN764198	-	USA
*Mycenalonginqua*	BAP 648	MH414552	MH385328	Príncipe
*Mycenamaculata*	CBS 237.47	MH856232	MH867761	France
*Mycenamaculata*	CBS 239.47	MH856234	MH867763	France
*Mycenameliigena*	39	JF908423	-	Italy
*Mycenameliigena*	39d	JF908429	-	Italy
*Mycenametata*	iNat29471261	OK346496	-	USA
*Mycenapearsoniana*	TENN61384	JN182200	-	USA
*Mycenapearsoniana*	TENN61544	JN182199	-	USA
*Mycenaphaeonox*	BAP 615	MH414564	MH385338	São Tomé
*Mycenaplumbea*	PBM 2718 (CUW) Colorado	DQ494677	-	-
*Mycenapterigena*	AH56033	OQ633196	-	Spain
*Mycenapterigena*	H6038561	MW540701	-	Finland
*Mycenapura*	TENN65043	JN182202	-	-
*Mycenarosella*	Champ-21	KX449424	-	France
*Mycenarosella*	983a	JF908488	-	Italy
*Mycenaseminau*	ACL136	KF537250	-	Malaysia
*Mycenaseminau*	ACL308	KF537252	-	Malaysia
*Mycenasemivestipes*	TENN61770	FJ596888	-	USA
*Mycenastrobilinoidea*	151c	JF908392	-	Italy
*Mycenastrobilinoidea*	151e	JF908393	-	Italy
*Mycenastrobilinoidea*	NX0647	MG654743	-	China
*Mycenastrobilinoidea*	NX0648	MG654744	-	China
*Mycenasubcaerulea*	TENN-F-051121	OL711671	OL711666	USA
*Mycenasubcaerulea*	TENN-F-057919	OL711672	OL711667	USA
*Mycenasupina*	128a	JF908388	-	Italy
*Mycenatenax*	OSC 113728	EU669224	-	USA
*Mycenatenax*	OSC 113746	EU846251	-	USA
*Mycenatubariodes*	H6039061	MW540704	-	Finland
*Mycenavulgaris*	447h	JF908435	-	Italy
*Mycenaxantholeuca*	LE 321752	MK474933	-	Russia
*Phloeomanaminutula*	H6036841	MW540684	-	Finland
*Phloeomanaminutula*	iNAT: 100003394	ON206666	-	USA
